# Supplementary material for: Cooperativity of c-MYC with Krüppel-Like Factor 6 Splice Variant 1 induces phenotypic plasticity and promotes prostate cancer progression and metastasis
Source: bioRxiv. 2024 Feb 1:2024.01.30.577982. Preprint. [Version 1] doi: 10.1101/2024.01.30.577982 (PMC10862900; doi:10.1101/2024.01.30.577982)

bioRxiv preprint doi: https://doi.org/10.1101/2024.01.30.577982; this version posted February 1, 2024. The copyright holder for this preprint (which was not certified by peer review) is the author/funder, who has granted bioRxiv a license to display the preprint in perpetuity. It is made available under aCC-BY 4.0 International license.

Supplemental Figures

Figure S1, Related to Figure 1. MYC-SV1 tumors are poorly differentiated carcinomas and locally invasive. A, Hematoxylin and Eosin (H&E) staining of MYC-SV1 mouse prostate. **B,** An invasive adenocarcinoma lesion in a MYC-SV1 mouse exhibiting invasion into the bladder (yellow arrow), periprostatic adipose (blue arrow), muscle (orange arrow), and prostatic capsule (green arrow). **C,** Representative image of smooth muscle actin (SMA) staining (green fluorescence) and DAPI nuclear staining (blue fluorescence). Blood vessels serve as a positive internal control (10x, scale bar=100 µm).

Figure S2, Related to Figure 2. Androgen and p63 expression in GEMMs. A, Strong nuclear AR is expressed in all mouse models. **B,** Loss of p63 is observed in tumors from MYC-SV1 mice. **C,** Molecular characterization by IHC staining with prostate lineage-marker specific antibodies on consecutive sections of a large poorly differentiated MYC-SV1 prostate carcinoma with un-involved well-differentiated adjacent adenocarcinoma (5x magnification, scale bar=600µm) for H&E, c-MYC, KLF6-SV1, AR, E-cadherin, p63, PCNA, Caspase-3, and CD31 with purple arrows point to blood vessels within the tumor.

Figure S3, related to Figure 2. MYC-SV1 metastatic nodules are of epithelial origin. Expression of E-cadherin, Pan-CK, and alpha-smooth muscle actin.

Figure S4, Related to Figure 1. MYC-SV1 tumor kinetics. A, Timeline of MYC-SV1 mPIN and prostate cancer development and progression. **B**, Gross and

bioRxiv preprint doi: https://doi.org/10.1101/2024.01.30.577982; this version posted February 1, 2024. The copyright holder for this preprint (which was not certified by peer review) is the author/funder, who has granted bioRxiv a license to display the preprint in perpetuity. It is made available under aCC-BY 4.0 International license.

photomicrographs of FFPE sections stained with hematoxylin and eosin (H&E) of tumor development kinetics of the mouse urogenital system. Scale bar = 1cm. **C,** Early stages of tumor development in MYC-SV1 mice. mPIN (blue arrow); adenocarcinoma and invasion (black arrow). **D**, Expression of pan-cytokeratin.

Figure S5, related to Figure 2. Histopathological presentation of hydronephrosis in MYC-SV1 mice. Representative gross and H&E-stained kidney sections from Hi-Myc and MYC-SV1 kidneys reveal hydronephrosis (arrow) in MYC-SV1 mice.

Figure S6, related to Figure 2. MYC-SV1 tumors metastasize to the liver and lymph node. Representative H&E stained FFPE sections of prostate cancer metastases to the liver and lymph nodes. Lack of smooth muscle actin and collagen expression (blue color) through Masson's Trichrome staining confirmed that the metastatic foci were not of myoepithelial or fibroblast origin.

Figure S7, related to Figure 4. Ingenuity Pathway Analysis of Hi-Myc vs. MYC-SV1 tumors. Ingenuity Pathway Analysis (IPA) was utilized to elucidate the global implications of the differentially expressed proteins in our four mouse cohorts. The ILK pathway emerged as a top canonical pathway associated with our proteomics data. Changes in differential protein expression are depicted in red (upregulated) and green (downregulated). Proteins identified within this pathway included F-Actin, HIC5, and vimentin.

Figure S8, related to Figure 7. *RNAish* and IHC staining of positive control specimens for KLF6-SV1 and c-MYC. A, Human prostate tissue stained with control probes. **B,** Human lymph node tissue stained for c-MYC protein (left). MYC-SV1 mouse

bioRxiv preprint doi: https://doi.org/10.1101/2024.01.30.577982; this version posted February 1, 2024. The copyright holder for this preprint (which was not certified by peer review) is the author/funder, who has granted bioRxiv a license to display the preprint in perpetuity. It is made available under aCC-BY 4.0 International license.

prostate stained for KLF6-SV1 protein (right).

Figure S1, related to Figure 1.

bioRxiv preprint doi: https://doi.org/10.1101/2024.01.30.577982; this version posted February 1, 2024. The copyright holder for this preprint (which was not certified by peer review) is the author/funder, who has granted bioRxiv a license to display the preprint in perpetuity. It is made available under aCC-BY 4.0 International license.

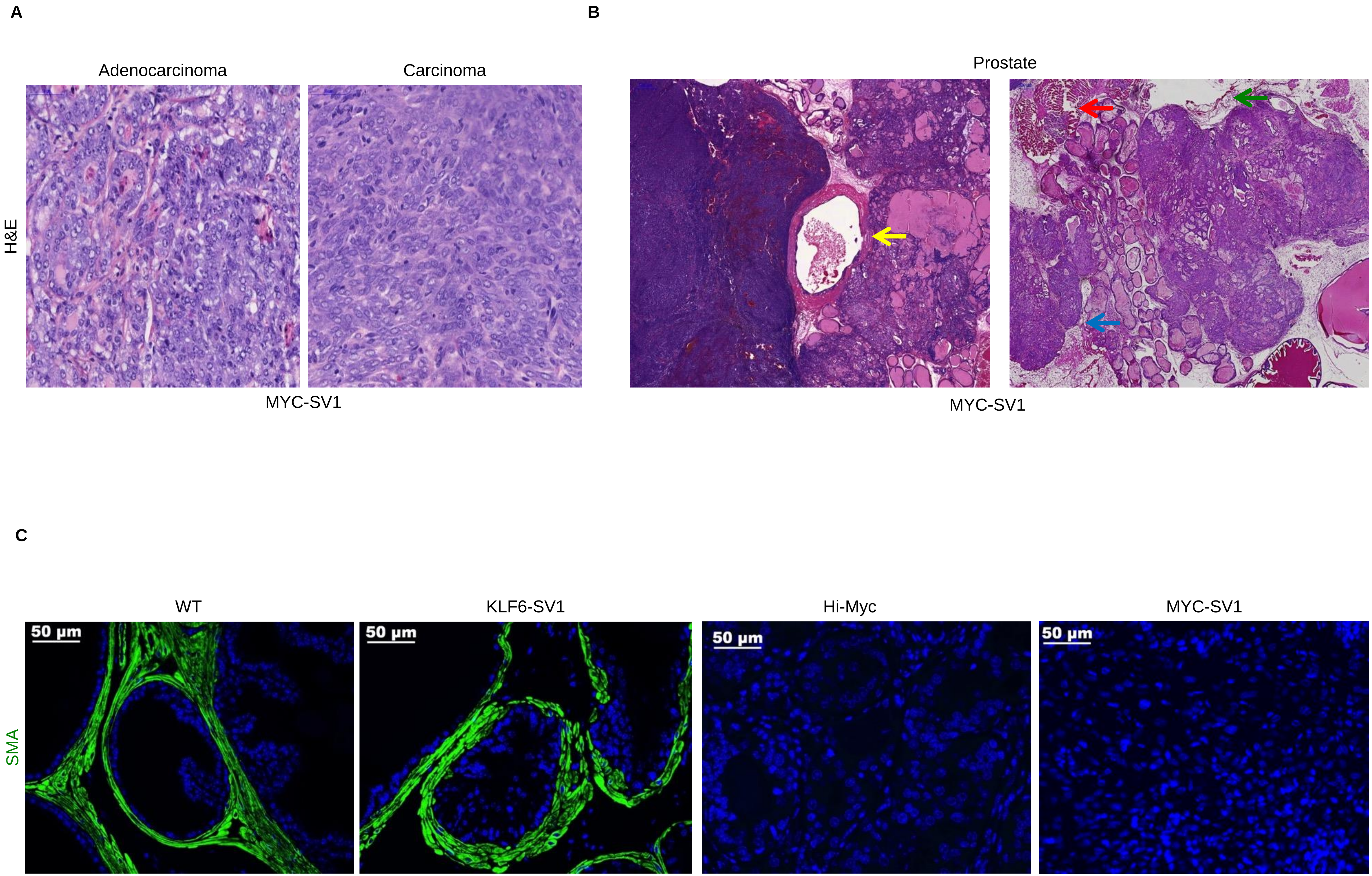

Figure S2, related to Figure 1.

bioRxiv preprint doi: https://doi.org/10.1101/2024.01.30.577982; this version posted February 1, 2024. The copyright holder for this preprint (which was not certified by peer review) is the author/funder, who has granted bioRxiv a license to display the preprint in perpetuity. It is made available under aCC-BY 4.0 International license.

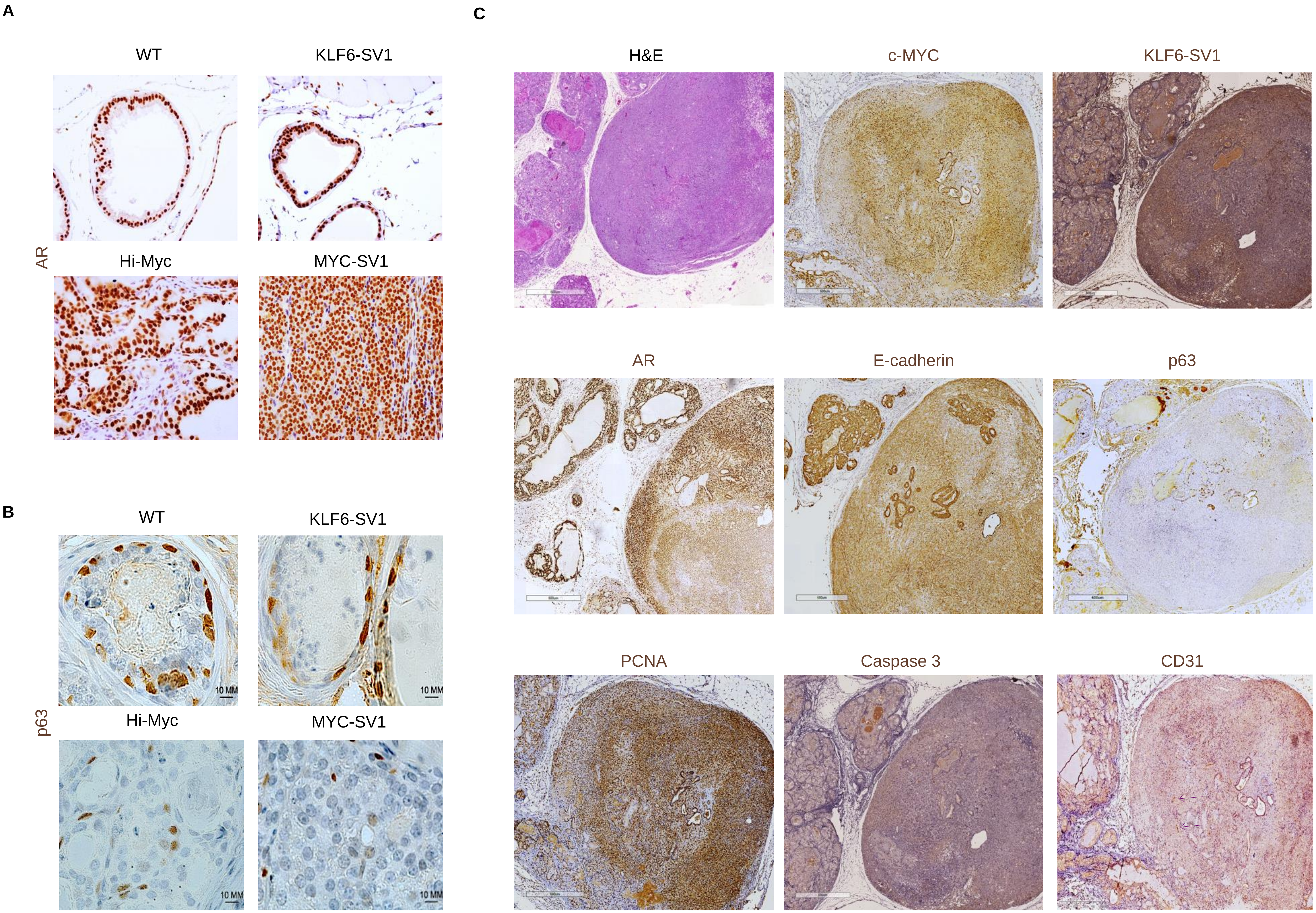

Figure S3, related to Figure 2.

bioRxiv preprint doi: https://doi.org/10.1101/2024.01.30.577982; this version posted February 1, 2024. The copyright holder for this preprint (which was not certified by peer review) is the author/funder, who has granted bioRxiv a license to display the preprint in perpetuity. It is made available under aCC-BY 4.0 International license.

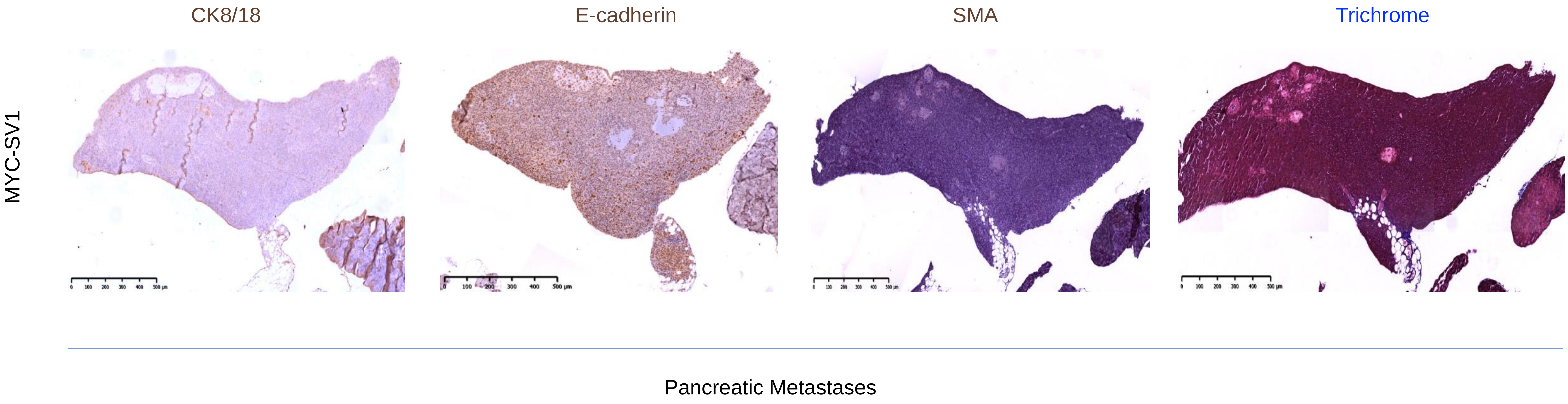

Figure S4. Related to Figure 2.

bioRxiv preprint doi: https://doi.org/10.1101/2024.01.30.577982; this version posted February 1, 2024. The copyright holder for this preprint (which was not certified by peer review) is the author/funder, who has granted bioRxiv a license to display the preprint in perpetuity. It is made available under aCC-BY 4.0 International license.

A

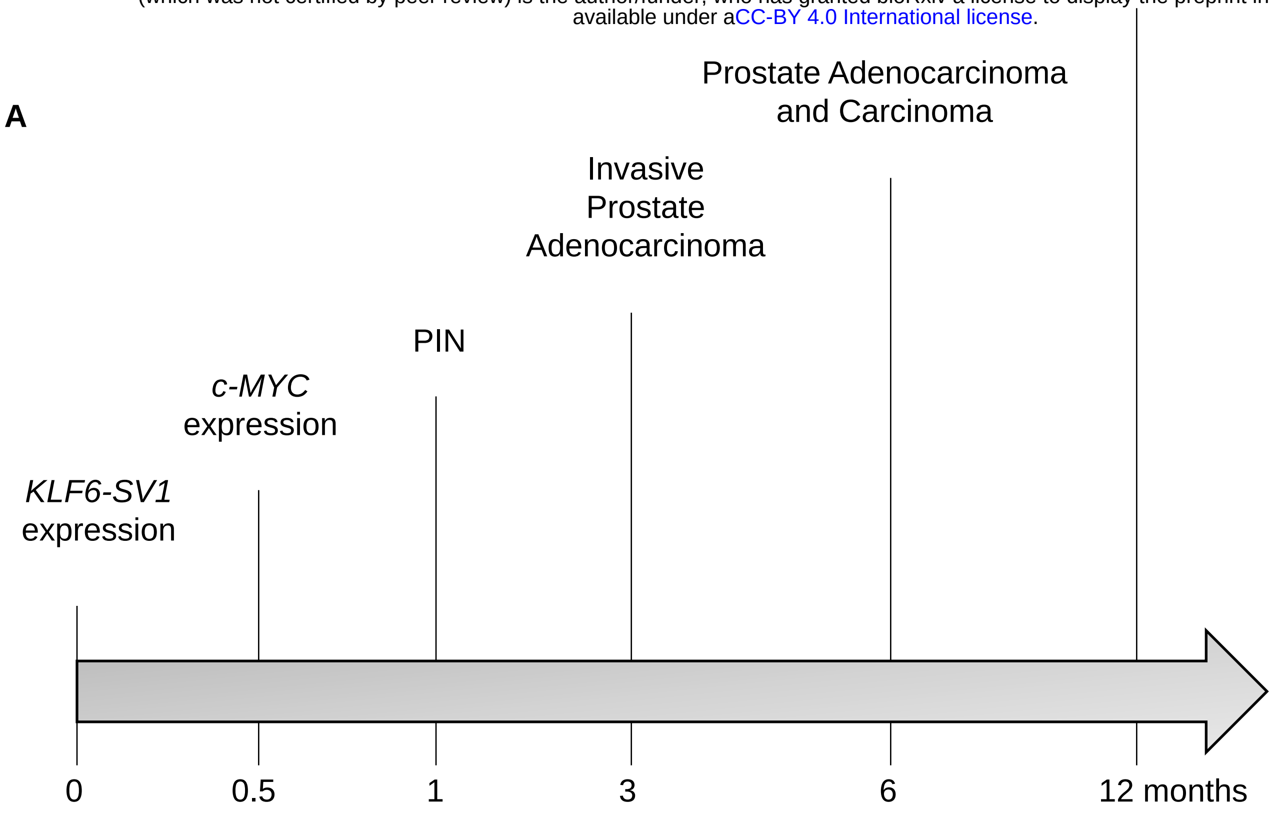

B

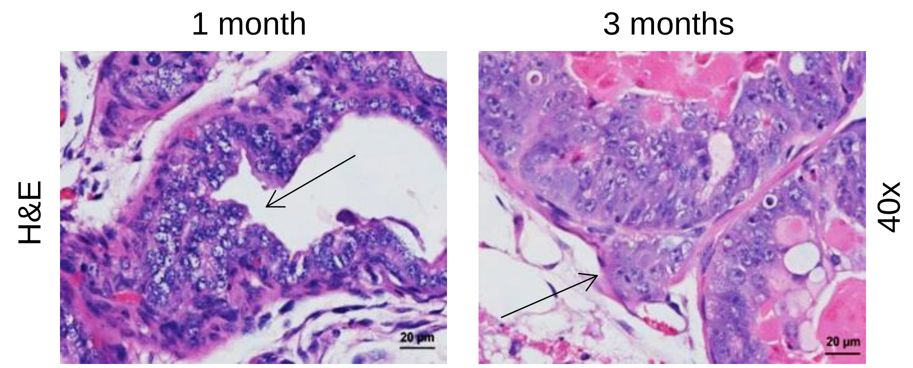

MYC-SV1	Phenotype (n)	Invasion (n)
1 month	PIN (3/3)	No (0/3)
3 months	Adenocarcinoma (4/4)	Yes (2/4)

C

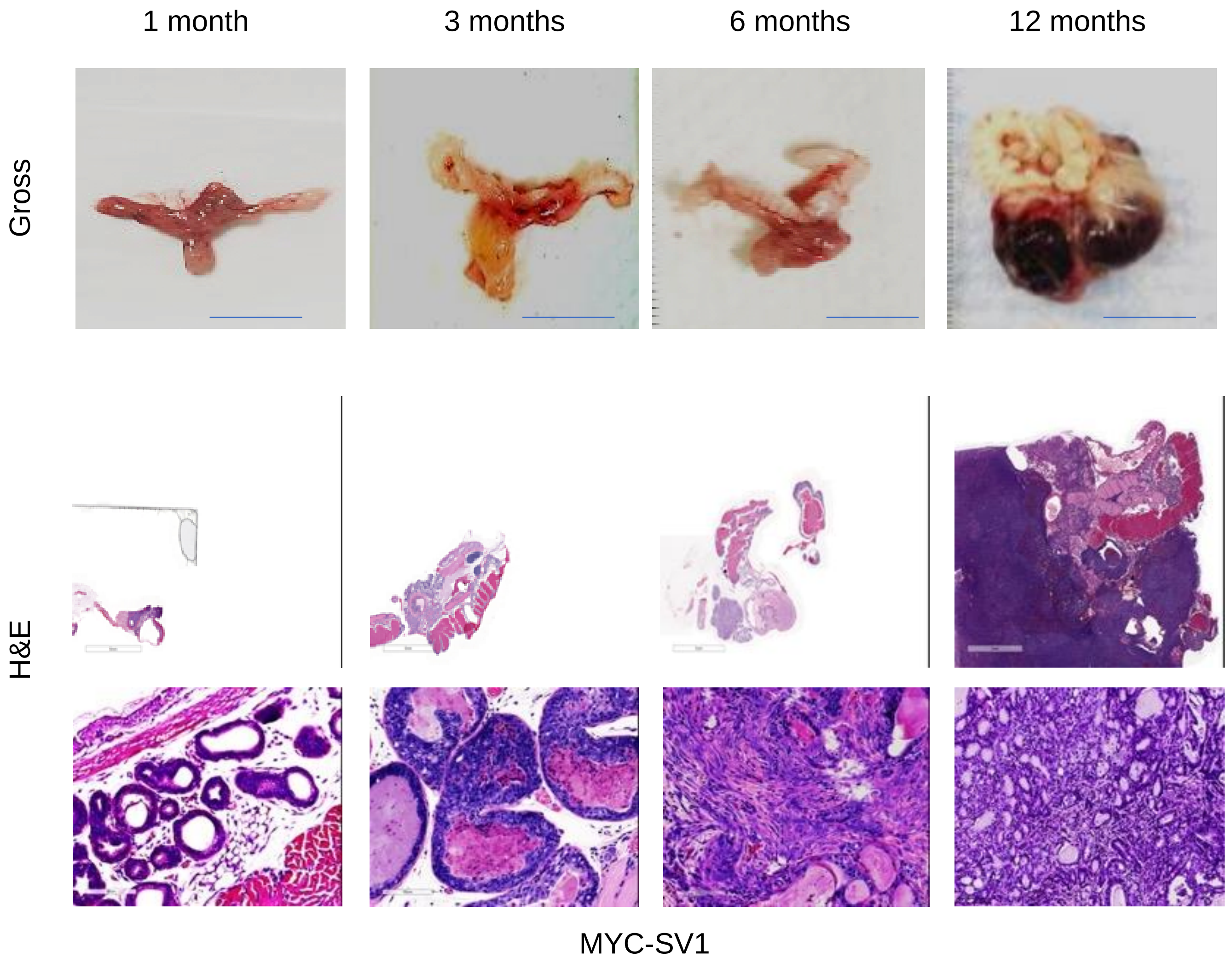

D

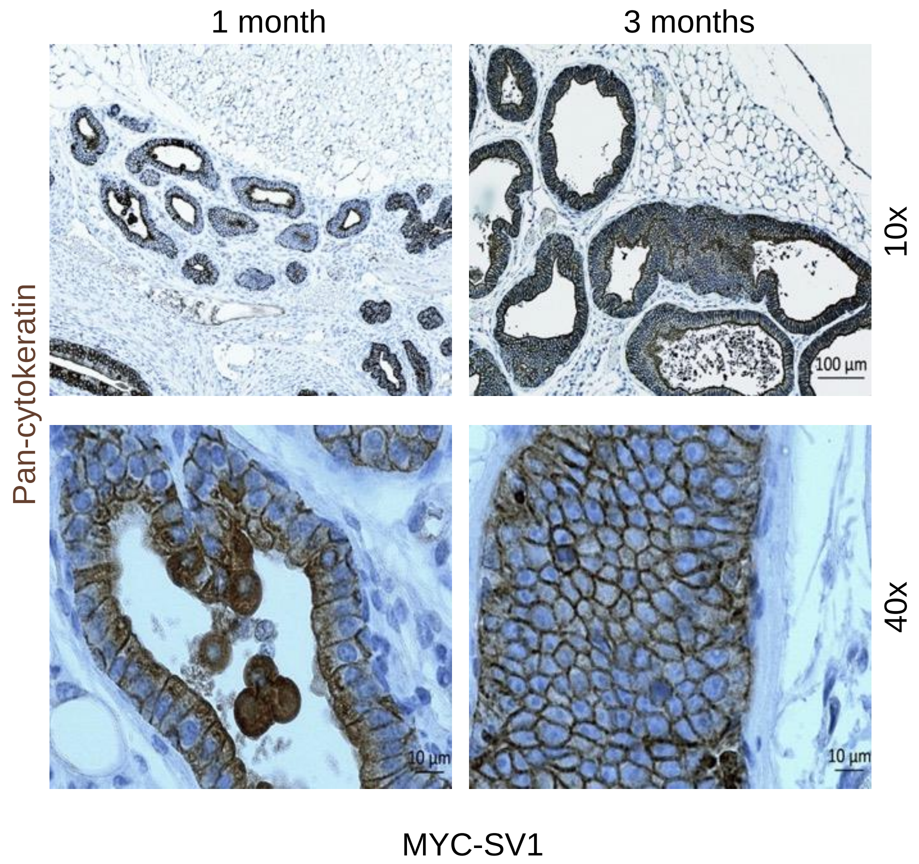

bioRxiv preprint doi: https://doi.org/10.1101/2024.01.30.577982; this version posted February 1, 2024. The copyright holder for this preprint (which was not certified by peer review) is the author/funder, who has granted bioRxiv a license to display the preprint in perpetuity. It is made available under aCC-BY 4.0 International license.

Figure S5. related to Figure 2.

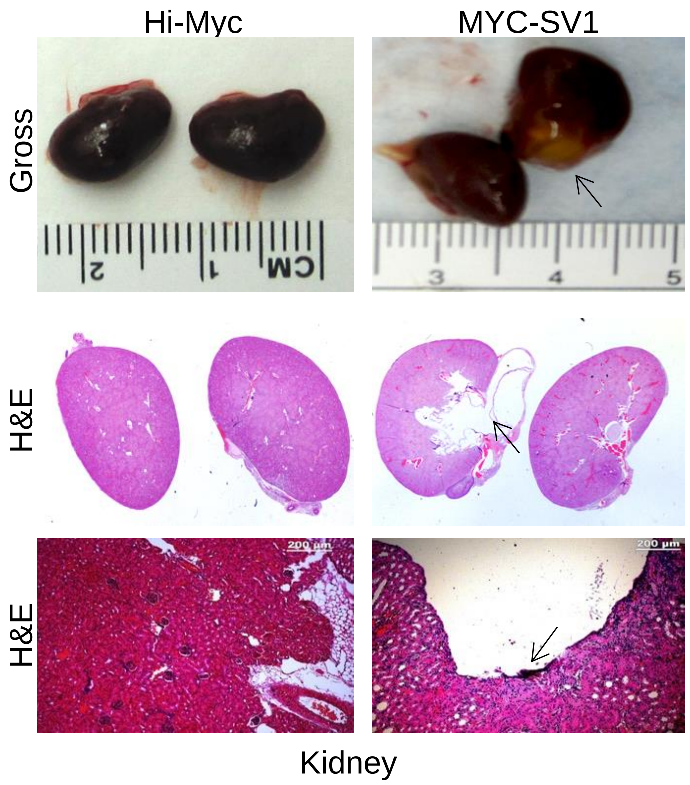

bioRxiv preprint doi: https://doi.org/10.1101/2024.01.30.577982; this version posted February 1, 2024. The copyright holder for this preprint (which was not certified by peer review) is the author/funder, who has granted bioRxiv a license to display the preprint in perpetuity. It is made available under aCC-BY 4.0 International license.

Figure S6. Related to Figure 2.

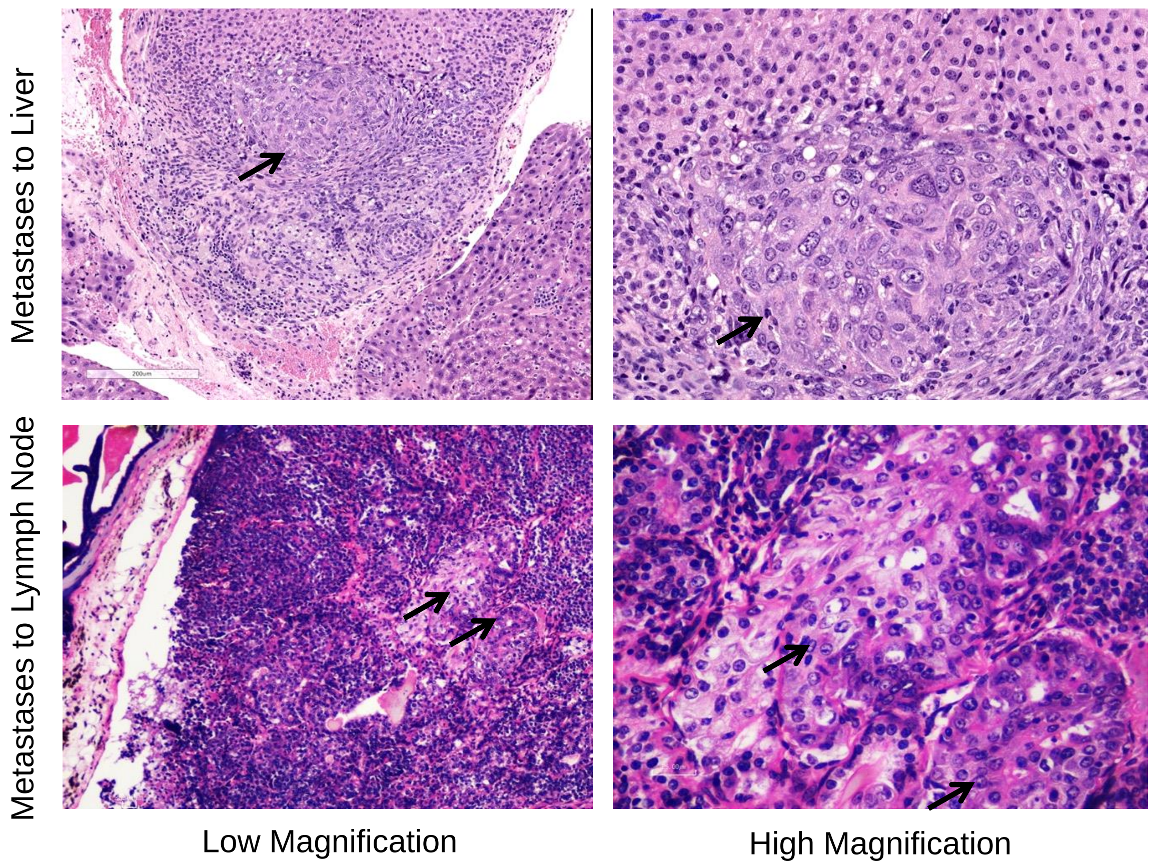

bioRxiv preprint doi: https://doi.org/10.1101/2024.01.30.577982; this version posted February 1, 2024. The copyright holder for this preprint (which was not certified by peer review) is the author/funder, who has granted bioRxiv a license to display the preprint in perpetuity. It is made available under aCC-BY 4.0 International license.

Figure S7. related to Figure 5.

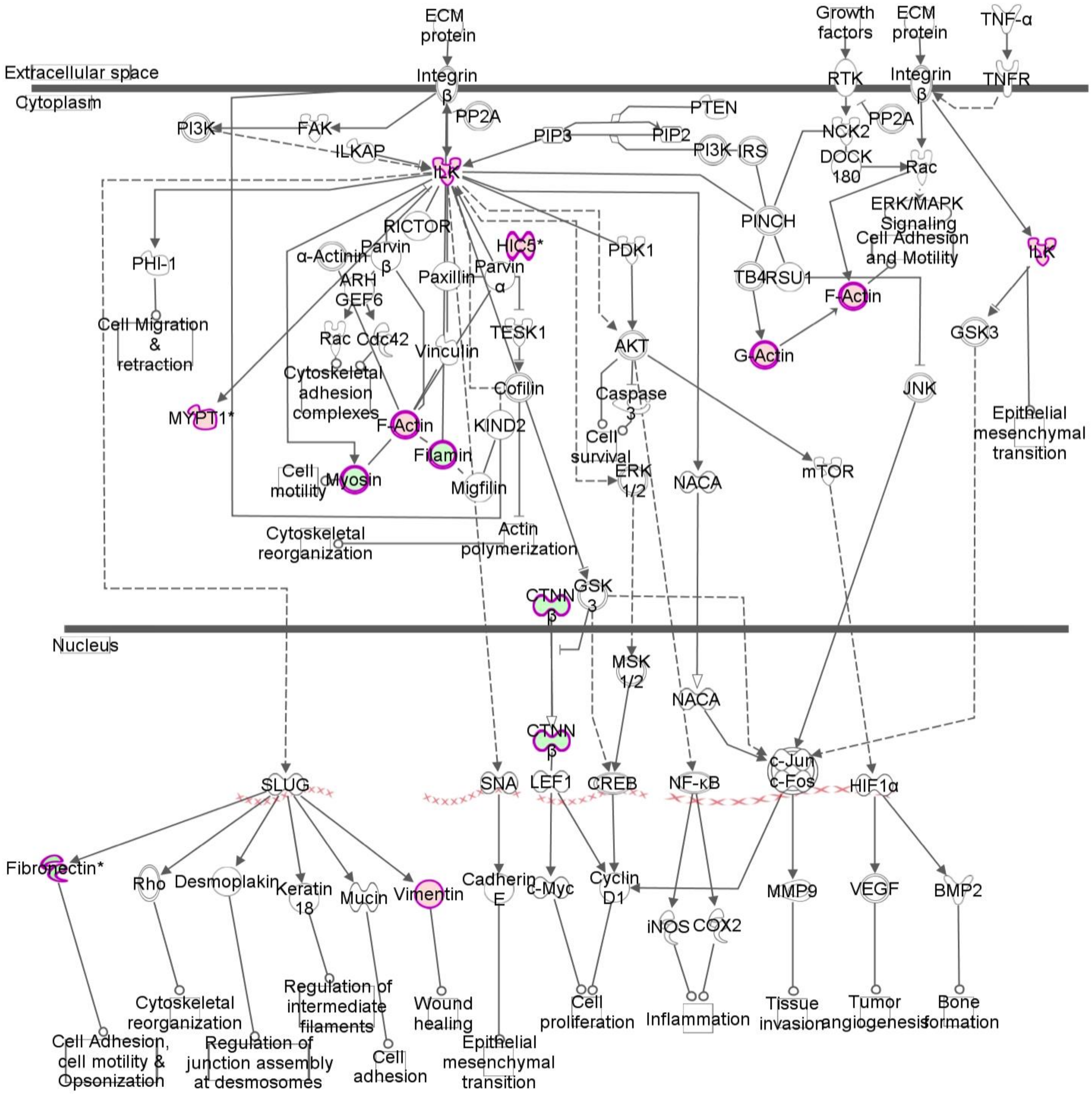

bioRxiv preprint doi: https://doi.org/10.1101/2024.01.30.577982; this version posted February 1, 2024. The copyright holder for this preprint (which was not certified by peer review) is the author/funder, who has granted bioRxiv a license to display the preprint in perpetuity. It is made available under aCC-BY 4.0 International license.

Figure S8, related to Figure 7.

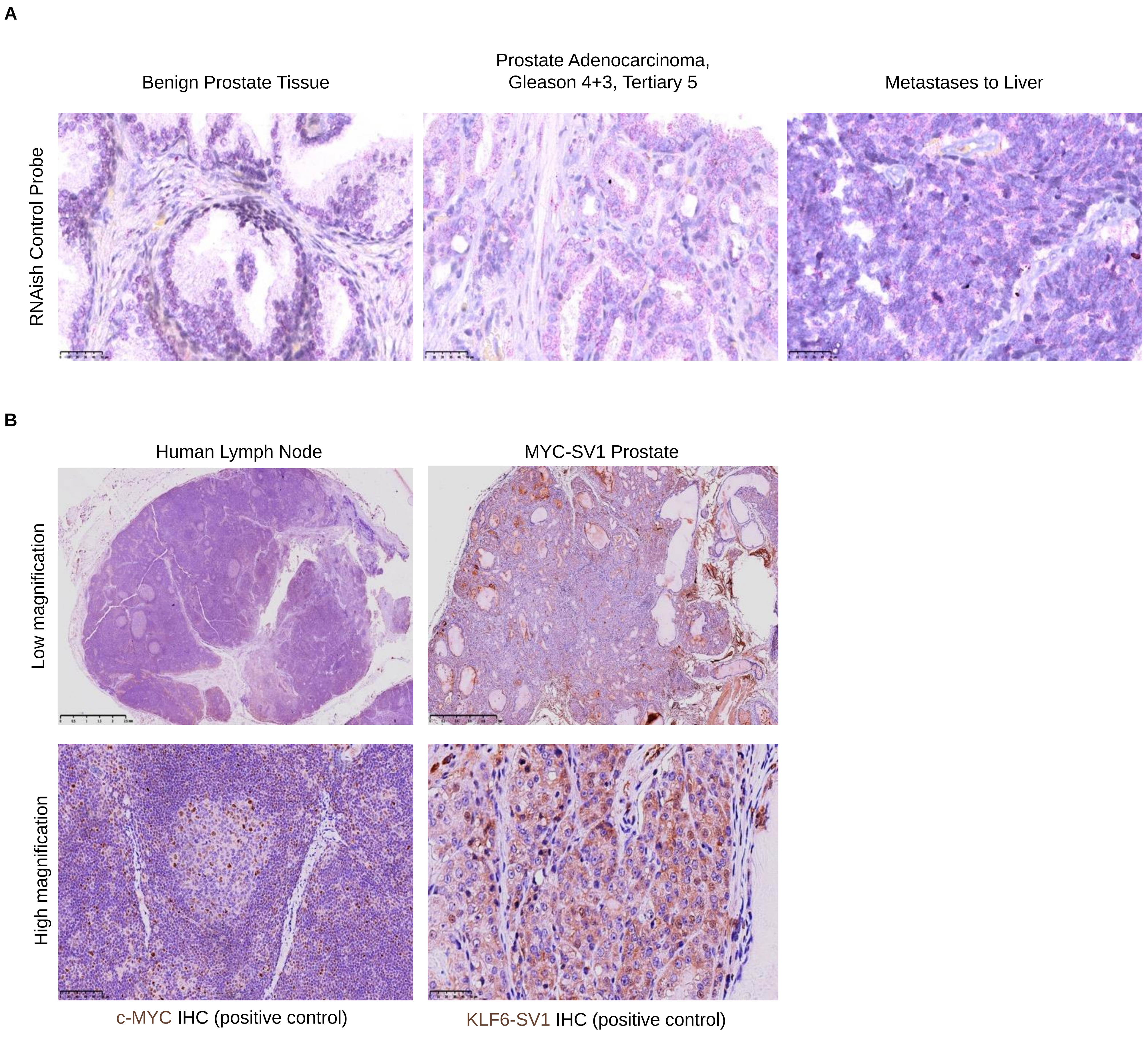

Supplement: Supplement 1 [file NIHPP2024.01.30.577982v1-supplement-1.pdf]
